# Parent-child discrepancies in the report of adolescent emotional and behavioral problems in Taiwan

**DOI:** 10.1371/journal.pone.0178863

**Published:** 2017-06-23

**Authors:** Ying-Yeh Chen, Suk-Yin Ho, Pei-Chen Lee, Chia-Kai Wu, Susan Shur-Fen Gau

**Affiliations:** 1 Taipei City Psychiatric Center, Taipei City Hospital, Taipei, Taiwan; 2 Institute of Public Health and Department of Public Health, National Yang-Ming University, Taipei, Taiwan; 3 Child Developmental Assessment & Intervention Center, Taipei City Hospital, Taipei, Taiwan; 4 National Taipei University of Nursing and Health Sciences, Taipei City, Taiwan; 5 Department of Psychiatry, National Taiwan University Hospital and College of Medicine, Taipei, Taiwan; 6 Graduate Institute of Brain and Mind Sciences, College of Medicine, National Taiwan University, Taipei, Taiwan; 7 Department of Psychology, School of Occupational Therapy, Graduate Institute of Epidemiology and Preventive Medicine, National Taiwan University, Taipei, Taiwan; Radboud Universiteit, NETHERLANDS

## Abstract

The majority of studies on parent-child discrepancies in the assessment of adolescent emotional and behavioral problems have been conducted in Western countries. It is believed that parent-adolescent agreement would be higher in societies with a strong culture of familism. We examined whether parent-adolescent discrepancies in the rating of adolescent emotional and behavioral problems are related to parental and family factors in Taiwan. Participants included 1,421 child-parent pairs of 7^th^-grade students from 12 middle schools in Northern Taiwan and their parents. We calculated Pearson’s correlation coefficients to assess the relationship between parental (Child Behavior Checklist, CBCL) and adolescent (Youth Self Report, YSR) report of emotional/behavioral problem syndromes. Regression models were used to assess parent-adolescent differences in relation to parental psychopathology and family factors. We found that parent-adolescent agreement was moderate (*r* = 0.37). Adolescents reported higher symptom scores than their parents (Mean Total Problem Score: CBCL: 20.79, YSR: 33.14). Parental psychopathology was related to higher parental ratings and better informant agreement. Parents with higher socioeconomic status (SES) tended to report lower scores for adolescent problem syndromes, resulting in higher levels of disagreement. Greater maternal care was related to higher parent-adolescent agreement. Based on our study findings, we conclude that familism values do not seem to improve parent-child agreement in the assessment of adolescent problem syndromes. The finding that higher SES was related to increased discrepancies speaks to the need to explore the culture-specific mechanisms giving rise to informant discrepancies.

## Introduction

Disagreement between self and parental report of adolescent psychopathology is common [1–4]. A recent meta-analysis of 341 studies conducted between 1989 and 2014 revealed a low-to-moderate correspondence between informant reporting of children’s mental health concerns [5]. These discrepancies pose major challenges for clinical practice, research, and theory related to child psychiatry and psychology.

The most commonly proposed factor contributing to the observed parent-child discrepancy is the type of disorder being observed; there are indications that agreement between child and parental reporting is stronger with respect to externalizing than internalizing symptoms [5–7]. Externalizing behaviors, such as aggression and hyperactivity, are more likely to be observed directly, whereas internalizing symptoms, such as anxiety and depression, are less observable in nature. Although the proposition that externalizing symptoms yield greater agreement has been supported by several previous investigations [8–11], one recent systematic comparison of 25 societies by Rescorla et al. did not find differences in the level of parent-child agreement for internalizing and externalizing symptoms [12].

Apart from the overtness of the children’s symptoms, family factors such as parental level of psychopathology, parental socioeconomic status (SES), family structure, and family conflicts are also related to informant discrepancies [5, 13]. It is hypothesized that depressive informants may report a greater level of concern for their children’s mental health relative to reports taken from other informants [10, 13–16]. However, the empirical support for this hypothesis is mixed; some studies have found no such support [17–19]. Relatively little attention has been paid to the association between family characteristics and informant discrepancies [10, 13, 15, 16]. Existing studies, although few, tend to suggest that child and parent discrepancies are greater in families with lower SES, single-parent families, households with a large number of children, and families with greater parent-child conflict [13, 15, 16, 20]. These family characteristics may be associated with inadequate parent-child communication, and hence, may result in lower agreement in their reports.

Some researchers have hypothesized that parent-child discrepancies may be smaller in societies where cultural values promote familism and collectivism, such as those with Confucian traditions in East Asia [21]. Asian culture emphases ‘interdependence’, in contrast to ‘independence’ practiced in many Western countries [22]. Chinese parenting is expected to be highly involved, responsible for decision making, and caring for children throughout their lives [22, 23]. Interdependence-based family relationships may improve parent-child agreement in the assessment of adolescents psychopathology since social values, life perspectives, and decision making are readily shared in Chinese families. However, to the best of our knowledge, there have been very few studies from East Asian countries that specifically explore the relationships between family characteristics and parent-child agreement in the report of adolescent emotional and behavioral symptoms. One study from China has demonstrated high parent-adolescent agreement (*r* = 0.6) on emotional and behavioral problems [20]. The study by Rescorla et al. found that the cross-informant correlations for China and Hong Kong were 0.46 and 0.41, respectively, and for two other Asian countries, Japan and South Korea, were 0.23 and 0.53, respectively [12].

To provide further information to this area of research, we used school-based survey data from 1,412 students aged 12–13 in Taiwan and assessed the degree of agreement between child and parental reports of adolescent emotional and behavioral problems. In addition, we explored whether family characteristics, including parental psychopathology, family structure, family SES, and maternal bonding, were associated with disagreement in parent-adolescent reporting of emotional and behavioral symptoms.

## Methods

### Participants

Participants were recruited from 6 of the 73 junior high schools in Taipei City, and 6 of the 66 in New Taipei City in 2003. The Taipei City and New Taipei City school districts were each categorized into three groups based on SES and neighborhood location. Two schools were randomly selected from each group using simple randomization procedure. All the students in the 36 selected classes were recruited to generate an eligible population of 1,518 students aged 12 to 13 years. All the parents of the recruited students were invited to complete a parental questionnaire. Approximately 67% of the parental questionnaire was filled in by mothers, and 33% was reported by fathers. The parents of 91 students did not turn in the questionnaire. We further excluded those who did not provide complete data on our outcome measures. The final sample size used in the current analysis was 1,412 parent-adolescent pairs. This study was approved by the Research Ethics Committee of the National Taiwan University Hospital, Taipei, Taiwan (Approval ID, 9100002328).

### Measures

#### 1) Adolescent emotional/behavioral problem symptoms

The Chinese Versions of the Child Behavioral Checklist (CBCL) reported by the parental participants, and the Youth Self-Report (YSR) reported by the adolescents were used to assess adolescent emotional/behavioral problem syndromes. The CBCL is a parental questionnaire used to measure the parental perception of children and adolescents aged 4 to 18 [24]. The YSR is administered to adolescents aged 11–18 to obtain self-reports about their problem syndromes [24]. The Chinese versions of the CBCL and YSR used in Taiwan were based on the 1991 versions [24]; their development is detailed elsewhere [25–27]. Briefly, the Chinese versions were prepared via a two-stage translation with the permission and approval of the original author [25, 28]. The majority of the items on the YSR are generally equivalent to the CBCL, but are worded in the first person; nonequivalent items were eliminated from the current analysis. A total of 83 items common to both forms were retained to create eight narrow-band behavioral/emotional problem syndromes for both the CBCL and YSR, including attention problems, anxious/depressed symptoms, aggressive behaviors, delinquent behaviors, social problems, somatic complaints, thought problems, and withdraw. Each item was scored 0 if not true, 1 if somewhat or sometimes true, and 2 if very true or often true. S1 Table presents items of the Chinese version CBCL and YSR used in the current analysis with the corresponding items in English. Two broad-band groups of syndromes, designated as ‘Internalizing’ (including withdraw somatic complaints, and anxious/depressed) and ‘Externalizing’ (including delinquent behavior and aggressive behavior) were also generated to conduct the current analysis.

The reliability and validity of the Chinese versions of the YSR and CBCL have been demonstrated previously in Taiwan [25–27], and these two scales have been widely used in child and adolescent research in Taiwan [29, 30].

#### 2) Parental and family characteristics

**a) Parental psychopathology—Chinese Health Questionnaire (CHQ)**

The CHQ is a 12-item self-reporting questionnaire modified from the General Health Questionnaire [31]. The CHQ has been widely used to identify minor psychiatric disorders in both the primary care and community setting [32]. The cutoff point for cases was set at 4 and above [32].

**b) Family structure and family socioeconomic status (SES)**

Family structure was assessed by parental marital status (married vs. not currently married) and sibship size (single, two, and three or more). Family SES was measured by paternal educational attainment. A recent study from Taiwan has shown that paternal educational attainment is more sensitive than the maternal educational attainment in predicting mental health outcomes [33].

**c) Maternal bonding—Chinese version of the Parental Bonding Index (PBI)**

This instrument was administered to adolescents to report their perceived bonding with their mothers. The PBI is a 25-item instrument (rated on a four-point Likert scale from 1 [very likely] to 4 [very unlikely]) measuring parenting styles during the child’s first 16 years, using two principal dimensions—care (12 items) and protection (13 items). Higher scores on the care subscale reflect affection and warmth; lower scores indicate rejection or indifference. Higher scores on the protection scale indicate greater parental authoritarian control and overprotectiveness. The reliability and validity of the Chinese version of the PBI have been demonstrated elsewhere [34]. The Chinese PBI has been widely used in research in Taiwan [35].

#### 3) Control covariates

We controlled for adolescent gender and parental age (paternal age >50 vs. < = 50; maternal age > 45 vs. < = 45).

### Analytic strategies

We compared the eight emotional/behavioral problem syndromes and the internalizing and externalizing problems assessed by parental report of the CBCL and the adolescent report of the YSR. Pearson’s correlation coefficients (*r*) were presented for the correlation between the CBCL and YSR scores. Total scores for the CBCL and YSR in terms of internalizing and externalizing syndromes were compared according to different sociodemographic and family characteristics.

Mixed-model ANOVAs were used to test for mean differences between the corresponding original scores of the CBCL and YSR subscales. We used the Bonferroni correction to decrease the possibility of false positives due to multiple comparisons. The significance level was set at 0.0028 (i.e. 0.05/18 = 0.0028).

Multiple linear regression analyses were used to assess whether parental and family factors were related to differences in parental-adolescent agreement (YSR score minus CBCL score was treated as our dependent variable).

## Results

Table 1 presents the scores of each emotional and behavioral problem syndromes of the CBCL and YSR, their Pearson’s correlation coefficients, their score differences, and the 95% confidence intervals of the respective scores. The average emotional and behavioral problem syndrome scores reported by adolescents were higher than parental reports (Mean Total Problem Score: CBCL: 20.79, YSR: 33.14) (Table 1). The Pearson’s correlation coefficients between the CBCL and YSR were in the range of 0.25 and 0.40 (Table 1). The correlation coefficient for internalizing problems was the same as that for externalizing problems (0.36). The correlation coefficient for the total scores was 0.37.

**Table 1:** Parent-adolescent report of emotional and behavioral problem syndromes.

	Parental report	Adolescent report	r	Difference	t value	P value
	(CBCL)	(YSR)				
	Mean (SD)	Mean (SD)		Mean (95%CI)		
Aggressive behaviors	4.77 (4.70)	7.03 (5.04)	0.35	-2.26(-2.55,-1.97)	-15.26	< .0001
Anxious / Depressed	3.20 (3.64)	5.58 (4.69)	0.34	-2.38(-2.64,-2.13)	-18.37	< .0001
Attention Problems	4.20 (3.39)	5.87 (3.38)	0.37	-1.68(-1.87,-1.48)	-16.62	< .0001
Delinquent behavior	1.35 (1.91)	2.77 (2.46)	0.33	-1.42(-1.55,-1.29)	-20.79	< .0001
Social Problems	2.27 (2.17)	3.01 (2.27)	0.40	-0.74(-0.87,-0.61)	-11.39	< .0001
Somatic Complaints	1.71 (2.33)	3.15 (3.10)	0.35	-1.45(-1.61,-1.28)	-17.18	< .0001
Thought Problems	0.84 (1.26)	1.82 (1.87)	0.25	-0.98(-1.08,-0.88)	-18.73	< .0001
Withdrawn	2.45 (2.41)	3.89 (2.75)	0.30	-1.44(-1.60,-1.28)	-17.66	< .0001
Total problems	20.79(17.45)	33.14(20.16)	0.37	-12.35(-13.46,-11.24)	-21.79	< .0001
Internalizing problems	7.35 (7.22)	12.62 (9.17)	0.36	-5.27(-5.76,-4.78)	-21.01	< .0001
Externalizing problems	6.13 (6.23)	9.80 (6.99)	0.36	-3.68(-4.07,-3.29)	-18.39	< .0001

CBCL: Child Behavioral Checklist, YSR: Youth Self-Report

Table 2 illustrates parent-adolescent agreement on the CBCL/YSR scores for internalizing/externalizing problems based on different sociodemographic characteristics, CHQ, and maternal bonding scores. Overall, adolescents reported higher symptom scores than their parents, regardless of their basic demographic and family characteristics. In addition, girls reported higher levels of internalizing problems than boys; whereas boys reported higher levels of externalizing problems than girls. Interestingly, the corresponding parental reports were in the same direction. Older fathers tended to report lower symptom scores (in both internalizing and externalizing problems) than younger fathers, but their children tended to report greater levels of symptoms, resulting in greater discrepancies in this group. This pattern was similar for older mothers and their reporting of internalizing symptoms. Internalizing and externalizing problem scores (in both the parental and adolescent report) were found to be lower in integrated families, families of higher SES, and families with higher levels of maternal care. Parental psychopathology was associated with higher symptom scores in both parental and adolescent report.

**Table 2:** Parent-adolescent report of internalizing/externalizing problems in different sociodemographic groups.

	N(%)	Original score for Internalizing problems			Original score for Externalizing problems		
		Parental report	Adolescent report	*P* value^#^	Parental report	Adolescent report	*P* value^#^
		Mean (SD)	Mean (SD)		Mean (SD)	Mean (SD)	
**Sex of the child**				< .0001			< .0001
Male	702(49.72)	6.64(6.72)	10.83(8.25)		6.41(6.31)	10.20(7.14)	
Female	710(50.28)	8.06(7.61)	14.40(9.68)		5.85(6.14)	9.41(6.82)	
**Age**							
**Paternal age**				< .0001			< .0001
Paternal age >50	143(10.85)	6.95(6.99)	14.73(10.20)		5.84(6.10)	10.50(6.96)	
Paternal age < = 50	1175(89.15)	7.37(7.22)	12.30(8.96)		6.08(6.21)	9.66(6.93)	
**Maternal age**				< .0001			< .0001
Maternal age >45	265(19.73)	7.17(7.43)	14.61(9.69)		6.26(6.62)	10.62(6.79)	
Maternal age < = 45	1078(80.27)	7.35(7.16)	12.08(8.94)		5.97(6.03)	9.52(6.94)	
**Parental marital status**				< .0001			< .0001
Married	1229(88.48)	7.11(7.11)	12.37(9.00)		5.81(6.04)	9.55(6.88)	
Not married	160(11.52)	9.14(7.73)	14.26(10.20)		8.32(6.98)	11.47(7.69)	
**Sibship size**				< .0001			< .0001
Single child	124(8.88)	7.80(7.32)	12.65(9.73)		6.80(6.83)	10.45(7.90)	
Two	692(49.57)	7.00(6.83)	11.88(9.04)		5.68(5.88)	9.46(6.97)	
Three or more	580(41.55)	7.67(7.61)	13.42(9.10)		6.47(6.43)	10.00(6.76)	
**Paternal educational attainment**				< .0001			< .0001
Some high school or lower	319(24.26)	8.64(7.49)	13.43(9.50)		7.46(6.73)	10.29(7.44)	
High school graduate	435(33.08)	7.46(7.26)	12.27(8.80)		6.30(6.21)	9.69(6.62)	
Some college	255(19.39)	7.13(7.58)	12.38(9.05)		5.52(5.61)	9.85(7.09)	
College degree or higher	306(23.27)	5.85(6.04)	12.18(9.25)		4.47(5.52)	9.11(6.83)	
**Parental psychopathology**				< .0001			< .0001
Yes (CHQ> = 4)	226(16.12)	11.09(8.26)	14.73(10.40)		8.33(6.78)	11.19(7.89)	
No (CHQ<4)	1176(83.88)	6.64(6.78)	12.18(8.83)		5.69(6.03)	9.48(6.74)	
**Maternal bonding**							
**Care**				< .0001			< .0001
High care^+^	703(49.79)	6.66(6.47)	11.16(8.57)		5.23(5.49)	8.43(6.43)	
Low care^++^	685(49.35)	8.04(7.89)	14.19(9.55)		7.02(6.81)	11.24(7.27)	
**Overprotection**				< .0001			< .0001
High overprotection^+^	683(48.37)	7.34(6.97)	12.78(9.35)		6.06(5.93)	9.79(6.98)	
Low overprotection^++^	700(50.61)	7.41(7.52)	12.54(9.06)		6.23(6.57)	9.84(7.05)	

^#^ Mixed-model ANOVAs were used to test for mean differences between the corresponding original scores of the CBCL and YSR subscales.

^+^ above mean,

^++^ mean or lower

Notes: missing values—Paternal age, N = 94; Maternal age, N = 69; Parental Marital status, N = 23; Sibship size, N = 16; Paternal educational attainment, N = 97; Parental psychopathology, N = 10; Care, N = 24; Overprotection, N = 29

Bonferroni correction was used to decrease the possibility of false positives. The significance level was set at 0.0028 (i.e. 0.05/18 = 0.0028).

Table 3 presents the results of regression models assessing parent-adolescent discrepancies (YSR score minus CBCL score) that consider all the independent and control variables. A higher level of parental psychopathology was negatively associated with a higher level of parent-adolescent disagreement; this was due to higher scores in parental rating (i.e. higher parental CHQ was associated with higher CBCL, hence the value of YSR minus CBCL became smaller). Parents who had high CHQ scores tended to give higher ratings to their children’s internalizing and externalizing problems than their low CHQ counterparts (internalizing problems: 0.46 points higher, P < .01; externalizing problems: 0.21 points higher, P = 0.04). Higher family SES was associated with a greater level of parent-adolescent disagreement on internalizing and externalizing problems when compared to the lowest SES group (internalizing problems: 1.97 points higher, P = 0.02; externalizing problems 1.95 points higher, P < .01); this disagreement was related to lower parentally reported scores. Having controlled for all covariates, a higher level of maternal care was significantly associated with lower discrepancies in the reporting of internalizing and externalizing problems.

**Table 3:** Regression models to assess the relationships between family characteristics and parent-adolescent agreement in the report of internalizing/externalizing problems.

	Internalizing problems				Externalizing problems			
	Sex& age adjusted		Adjusting for family characteristics		Sex&age adjusted		Adjusting for family characteristics	
	*β* estimate	*P* value	*β* estimate	*P* value	*β* estimate	*P* value	*β* estimate	*P* value
**Sex of the child**								
Male	1		1		1		1	
Female	2.14	< .0001	2.15	< .0001	-0.27	0.52	-0.29	0.50
**Age**								
Paternal age < = 50	1		1		1		1	
Paternal age >50	1.48	0.12	1.42	0.15	0.51	0.50	0.41	0.61
Maternal age < = 45	1		1		1		1	
Maternal age >45	2.11	0.01	2.17	0.01	0.79	0.19	0.77	0.22
**Parental Psychopathology (CHQ)**			-0.46	< .001			-0.21	0.04
**Parental marital status**								
Married			1				1	
Not married			-0.49	0.64			-0.47	0.58
**Sibship size**								
Single child			1				1	
Two			-0.39	0.71			-0.37	0.65
Three or more			0.94	0.38			-0.07	0.93
**Paternal educational attainment**								
Some high school or lower			1				1	
High school graduate			0.10	0.88			0.73	0.21
Some college			0.44	0.60			1.20	0.08
College degree or higher			1.97	0.02			1.95	0.003
**Maternal PBI**								
Care			-0.14	< .001			-0.08	0.02
Over-protection			0.00	0.93			0.02	0.55

## Discussion

### Main findings

We found the correlation between the parent and child reporting of adolescent emotional and behavioral problem syndromes to be moderate in Taiwan (*r* = 0.37). Levels of the agreement did not vary substantially across different types of emotional and behavioral problem syndromes. As expected, parental psychopathology was related to higher parental ratings of adolescent problem syndromes. However, parents in high SES families tended to report lower scores for adolescent emotional and behavioral problem syndromes. In addition, better maternal bonding was found to be associated with slightly reduced informant discrepancies for both internalizing and externalizing problem syndromes.

### Interpretations and comparisons with previous research

#### 1) Cultural context and parent-adolescent agreement

We hypothesized that in a Confucian society like Taiwan, the stronger tradition of familism and collectivism would result in greater parent-adolescent agreement. Our results, however, do not support this proposition. The correlation for the total scale was 0.37, and none of the correlation coefficients for any dimensional problem assessed was greater than 0.4. This figure was much lower than a study conducted in China (r = 0.6) [20], but was quite similar to the cross-cultural study performed by Rescorla et al. (r = 0.46). Our mean Total Problem Syndrome scores were within 1 point of the Rescorla et al.’s estimations, which were based on 98 items in common [12].

Rescorla et al. also reported the correlation coefficients to be 0.53 in Korea, and 0.41 in Hong Kong; both were higher than the corresponding figure observed in the current study [12]. Nonetheless, they only found the CBCL-YSR correlation coefficient in Japan to be 0.23 [12]. Overall, existing findings seem to indicate that conventional dichotomies such as individualist vs. collectivist or Asian vs. Western do not appear to provide compelling explanations for the parent-child disagreement in the report of adolescent problem syndromes. Marked cultural variations were found within the Confucian/Collective cultural umbrella. For example, the strong ‘shame-culture’ in Japan (i.e. a deep cultural need for group approval and strong self-blame when violating social norms) [36] may have resulted in the higher symptom ratings among Japanese children (i.e. self-blame) and lower symptom ratings in Japanese parents (i.e. concealment of deviance) when compared to other Asian countries in the Rescorla et al. study [12]. The underlying social mechanisms leading to discrepancies in parent-child reporting may be highly nuanced, and not readily summarized by oversimplified cultural features.

Consistent with previous research results, adolescents tended to report higher levels of problems than their parents in the general population sample, which was particularly evident for internalizing problems such as anxiety and depression [12, 20, 37]. On the other hand, contrary results were observed in clinical samples, particularly for externalizing behavioral problem syndromes [38–41]. The parents in community samples might conservatively assess the problems of their children because they do not expect them to be unwell. This is not the case with a clinic-based sample of adolescents, who tend to underreport or even lie about their problems. Their parents, on the other hand, worry about their children’s conditions and thus are likely to observe their emotions and behaviors more closely. In addition, one recent study found somewhat better parent-adolescent agreement and more consistency in agreement patterns across diverse societies in clinical samples than in population samples [42]. Hence, factors affecting parent-child discrepancies in the assessment of children’s symptoms are context specific, with different perspectives, motivations, and attributions of the informants playing roles in information provision [43].

#### 2) Parental psychopathology and parent-child agreement

In line with previous studies, our current results indicate that parental psychopathology may cause the parent to encode children’s behaviors with greater negative descriptors [5, 10, 15]. However, rather than explaining this phenomenon as ‘depression-distortion,’ some researchers posit that parents with psychopathology may be more sensitive to children’s problems and hence the higher score rated by these parents may actually be more accurate [7, 44, 45]. Regardless of whether the higher parental ratings indicate accuracy or exaggeration, current findings suggest that parental psychopathology should be put into the equation when considering parent-adolescent discrepancies.

#### 3) Family characteristics and parent-child agreement

One important aim of this study was to explore the relationships between family characteristics and parent-child agreement. Very few previous studies have examined the impact of parental SES on informant discrepancies. One study reported no association between SES and parent-child discrepancy [46]. Another study reported that families on welfare tended to underestimate their children’s problems, whereas families not on welfare tended to relatively overestimate their children’s score [47]. Hypothetically, parents with higher SES are presumed to be more understanding of their children’s problems, and hence should produce evaluations more consistent with their children. Our results, however, were not in the expected direction. We found high family SES to be significantly correlated with comparatively low parental ratings of adolescent problems. This may imply that parents in higher SES do not expect their children to be unwell. Moreover, it is possible that the stigma attached to mental illness [48] may be related to the reluctance of ‘elite parents’ (i.e. those in high SES) to admit that their children have problems.

The association between greater maternal care and a lower level of disagreement in reporting was consistent with previous research. Previous studies, although few, have indicated that greater family cohesion [20] and higher parental acceptance [15, 16] are associated with greater consistency; whereas mother-child conflict [7, 14] and lack of communication [49] are related to the discrepancy. Although our construct—maternal bonding, may not be the same as the above mentioned constructs, they reflect very similar concepts, such as family function, cohesion, and warmth. Our study reveals that while the negative aspect of maternal bonding (i.e. overprotection) was not related to informant discrepancy, the positive aspect of bonding (i.e. care and warmth) was significantly, although slightly, associated with better reporting agreement. It is possible that caring mothers are more sensitive to their children’s problems. Hence, they may be more observant of their children’s problems.

We did not find a correlation between family structure (such as parental marital status and sibship size) and informant discrepancy when we controlled for all potential covariates. Previous studies on this association have been conflicting: one study showed that intact families and small family sizes were related to better consistency in parent-child reporting [46]; the other, like ours, did not detect this association [20].

### Implications

Even statistical consideration of several parent-child characteristics could not explain away informant disagreement in the family-oriented collective society of Taiwan. This resonates with the proposition of several researchers that disagreement among informants does not necessarily indicate unreliability in one or both informants; conversely, this may indicate that different informants contribute complementary information, and both are valid [7, 43, 50]. The best approach is to integrate reports from all informants, with discrepancies interpreted as informative, not problematic [7]. The meaning of the discrepancies should be explored in the context of the family background, parent-child relationships and respective characteristics of parents and children. An improved understanding of these dimensions would better inform intervention and treatment of the child’s psychopathology.

### Methodological considerations

This is one of few large-scale studies (N = 1,412) which has attempted to look into parent-adolescent disagreement in reporting emotional and behavioral problem syndromes in a non-Western setting. The current investigation responds to the call of several previous researchers and provides valuable empirical information which can be used to compare cross-informant data across multiple cultures [12, 51]. However, several limitations must be considered in the interpretation of our results. First, this is a cross-sectional survey and the observed relationships may not be causal. Second, all the measurements were obtained through self-report; no objective clinical assessments were conducted. Lastly, the sample was drawn from Taipei City and New Taipei City. Both of these locations are relatively urbanized areas in Taiwan which may further limit the generalizability of our findings to other parts of Taiwan and other Asian countries.

## Conclusion

We found moderate agreement in the parent-child reporting of adolescent emotional and behavioral problem syndromes in Taiwan. Our findings refute the hypothesis that societies with strong familism/collectivism traditions have greater parent-child agreement. Cultural attitudes of adolescent problematic behaviors should be further examined to understand the connotations underlying the assessment of each informant so that we can better help children who are suffering from different sets of problems.

## Supporting information

S1 Table Items of the Chinese version CBCL and YSR used in the current analysis with the corresponding items in English. (DOCX)

S1 Data Data used in the current analysis. (XLSX)
