# Supplementary material for: Parent-child discrepancies in the report of adolescent emotional and behavioral problems in Taiwan
Source: PLoS One. 2017 Jun 23;12(6):e0178863. doi: 10.1371/journal.pone.0178863 (PMC5482441; doi:10.1371/journal.pone.0178863)
Supplement: S1 Table — (DOCX) [file pone.0178863.s001.docx]

**S1 Table. Items of the Chinese version CBCL and YSR used in the current analysis with the corresponding items in English.**

| **Chinese version CBCL**  **0=不符合**  **1=部分符合**  **2=相當符合** | **CBCL**  **0=Not True (as far as you know)**  **1=Somewhat or Sometimes True**  **2=Very True or Often True** |
| --- | --- |
| 0 1 2 1. 表現得比他的實際年齡小  0 1 2 3. 好爭辯  0 1 2 7. 好吹牛、自誇  0 1 2 8. 不能長時間集中注意力  0 1 2 9. 無法停止想有些事情，強迫性思考  0 1 2 10. 坐不住、靜不下來，或活動量過高  0 1 2 11. 粘著大人或太依賴  0 1 2 12. 抱怨孤獨、寂寞  0 1 2 13. 思想、語言雜亂，或看起來頭腦不清的樣子  0 1 2 14. 好哭  0 1 2 16. 待人殘忍或卑賤，欺負弱小  0 1 2 17. 好做白日夢或沉溺於自己的思想中  0 1 2 19. 太要求別人的注意  0 1 2 20. 破壞自己的東西  0 1 2 21. 破壞家人或同伴的東西  0 1 2 22. 在家裡不守規矩  0 1 2 23. 在學校不守規矩  0 1 2 25. 不能跟其他小朋友和好相處  0 1 2 26. 犯錯後好像沒有罪惡感  0 1 2 27. 善妒  0 1 2 31. 怕自己可能會想或做不對的事  0 1 2 32. 覺得自己可能必須完美無缺  0 1 2 33. 覺得或抱怨沒有人愛他  0 1 2 34. 覺得別人對他有敵意  0 1 2 35. 自卑或覺得自己沒有價值  0 1 2 37. 常打架  0 1 2 38. 常被嘲笑  0 1 2 39. 常跟惹禍的同伴在一起  0 1 2 40. 聽到不存在的聲音  0 1 2 41. 衝動或不經考慮即行動  0 1 2 42. 喜歡獨處  0 1 2 43. 說謊或欺騙  0 1 2 45. 神經質或容易緊張  0 1 2 46. 出現緊張性動作或抽筋  0 1 2 48. 不被其他小孩喜歡  0 1 2 50. 過度害怕或焦慮  0 1 2 51. 覺得頭暈  0 1 2 52. 有過度的罪惡感  0 1 2 54. 過度疲倦  0 1 2 55. 體重過重  0 1 2 56. 有以下找不出生理原因的病痛：  0 1 2 a. 疼痛  0 1 2 b. 頭痛  0 1 2 c. 想吐，覺得身體不舒服  0 1 2 d. 與眼睛有關的問題  0 1 2 e. 發診或其他皮膚方面的問題  0 1 2 f. 胃吐或常說肚子痛  0 1 2 g. 嘔吐  0 1 2 57. 用身體攻擊他人  0 1 2 61. 功課不好  0 1 2 62. 動作協調不好或笨拙  0 1 2 63. 特別喜歡和年紀較大的孩子玩  0 1 2 64. 特別喜歡和年紀較小的孩子玩  0 1 2 65. 拒絕說話  0 1 2 66. 不斷重複某些動作，強迫性行為  0 1 2 67. 離家出走  0 1 2 68. 常大聲尖叫  0 1 2 69. 喜歡保守秘密，不希望別人知道他的事  0 1 2 70. 看到不存在的東西  0 1 2 71. 敏感，容易受窘  0 1 2 72. 縱火  0 1 2 74. 好賣弄或好扮小丑  0 1 2 75. 害羞或膽小  0 1 2 80. 發呆  0 1 2 81. 在家裡偷東西  0 1 2 82. 在外面偷東西  0 1 2 84. 怪異的行為  0 1 2 85. 怪異的念頭  0 1 2 86. 倔強固執，悶悶不樂、煩躁易怒  0 1 2 87. 情緒突然的轉變  0 1 2 88. 常鬧彆扭  0 1 2 89. 多疑  0 1 2 90. 咒罵或說髒話  0 1 2 93. 太多話或愛說話  0 1 2 94. 常嘲笑別人  0 1 2 95. 脾氣暴躁  0 1 2 96. 太常想到性  0 1 2 97. 恐嚇他人  0 1 2 102. 活動量低、動作緩慢、或無精打采  0 1 2 103. 不快樂、悲傷，或沮喪  0 1 2 104. 說話異常大聲  0 1 2 105. 非醫療用途而使用酒精或麻醉品  0 1 2 106. 有破壞物品的行為  0 1 2 112. 擔心、煩惱 | 0 1 2 1. Acts too young for his/her age  0 1 2 3. Argues a lot  0 1 2 7. Bragging, boasting  0 1 2 8. Can't concentrate, can't pay attention for long  0 1 2 9. Can't get his/her mind off certain thoughts; obsessions  0 1 2 10. Can't sit still, restless, or hyperactive  0 1 2 11. Clings to adults or too dependent  0 1 2 12. Complains of loneliness  0 1 2 13. Confused or seems to be in a fog  0 1 2 14. Cries a lot  0 1 2 16. Cruelty, bullying, or meanness to others  0 1 2 17. Day-dreams or gets lost in his/her thoughts  0 1 2 19. Demands a lot of attention  0 1 2 20. Destroys his/her own things  0 1 2 21. Destroys things belonging to his/her family or others  0 1 2 22. Disobedient at home  0 1 2 23. Disobedient at school  0 1 2 25. Doesn't get along with other kids  0 1 2 26. Doesn't seem to feel guilty after misbehaving  0 1 2 27. Easily jealous  0 1 2 31. Fears he/she might think or do something bad  0 1 2 32. Feels he/she has to be perfect  0 1 2 33. Feels or complains that no one loves him/her  0 1 2 34. Feels others are out to get him/her  0 1 2 35. Feels worthless or inferior  0 1 2 37. Gets in many fights  0 1 2 38. Gets teased a lot  0 1 2 39. Hangs around with others who get in trouble  0 1 2 40. Hears sounds or voices that aren't there  0 1 2 41. Impulsive or acts without thinking  0 1 2 42. Would rather be alone than with others  0 1 2 43. Lying or cheating  0 1 2 45. Nervous, highstrung, or tense  0 1 2 46. Nervous movements or twitching  0 1 2 48. Not liked by other kids  0 1 2 50. Too fearful or anxious  0 1 2 51. Feels dizzy  0 1 2 52. Feels too guilty  0 1 2 54. Overtired  0 1 2 55. Overweight  0 1 2 56. Physical problems without known medical cause:  0 1 2 a. Aches or pains (not headaches)  0 1 2 b. Headaches  0 1 2 c. Nausea, feels sick  0 1 2 d. Problems with eyes  0 1 2 e. Rashes or other skin problems  0 1 2 f. Stomach aches  0 1 2 g. Vomiting, throwing up  0 1 2 57. Physically attacks people  0 1 2 61. Poor school work  0 1 2 62. Poorly coordinated or clumsy  0 1 2 63. Prefers being with older kids  0 1 2 64. Prefers being with younger kids  0 1 2 65. Refuses to talk  0 1 2 66. Repeats certain acts over and over; compulsions  0 1 2 67. Runs away from home  0 1 2 68. Screams a lot  0 1 2 69. Secretive, keeps things to self  0 1 2 70. Sees things that aren't there  0 1 2 71. Self-conscious or easily embarrassed  0 1 2 72. Sets fires  0 1 2 74. Showing off or clowning  0 1 2 75. Shy or timid  0 1 2 80. Stares blankly  0 1 2 81. Steals at home  0 1 2 82. Steals outside the home  0 1 2 84. Strange behavior  0 1 2 85. Strange ideas  0 1 2 86. Stubborn, sullen, or irritable  0 1 2 87. Sudden changes in mood or feelings  0 1 2 88. Sulks a lot  0 1 2 89. Suspicious  0 1 2 90. Swearing or obscene language  0 1 2 93. Talks too much  0 1 2 94. Teases a lot  0 1 2 95. Temper tantrums or hot temper  0 1 2 96. Thinks about sex too much  0 1 2 97. Threatens people  0 1 2 102. Underactive, slow moving, or lacks energy  0 1 2 103. Unhappy, sad, or depressed  0 1 2 104. Unusually loud  0 1 2 105. Uses alcohol or drugs for nonmedical purposes  0 1 2 106. Vandalism  0 1 2 112. Worries |

| **Chinese version YSR**  **0=不符合**  **1=部分符合**  **2=相當符合** | **YSR**  **0=Not True (as far as you know)**  **1=Somewhat or Sometimes True**  **2=Very True or Often True** |
| --- | --- |
| 0 1 2 1. 表現得比我自己的實際年齡小  0 1 2 3. 好爭辯  0 1 2 7. 好吹牛、自誇  0 1 2 8. 不能長時間集中注意力  0 1 2 9. 無法停止想有些事情；  0 1 2 10. 坐不住、靜不下來，或活動量過高  0 1 2 11. 粘著大人或太依賴  0 1 2 12. 抱怨孤獨、寂寞  0 1 2 13. 思想、語言雜亂，或看起來頭腦不清的樣子  0 1 2 14. 好哭  0 1 2 16. 對人懷有敵意  0 1 2 17. 好做白日夢或沉溺於自己的思想中  0 1 2 19. 太要求別人的注意  0 1 2 20. 破壞自己的東西  0 1 2 21. 破壞家人或同伴的東西  0 1 2 22. 在家裡不守規矩  0 1 2 23. 在學校不守規矩  0 1 2 25. 不能跟其他小朋友和好相處  0 1 2 26. 犯錯後好像沒有罪惡感  0 1 2 27. 善妒  0 1 2 31. 怕自己可能會想或做不對的事  0 1 2 32. 覺得自己可能必須完美無缺  0 1 2 33. 覺得或抱怨沒有人愛我  0 1 2 34. 覺得別人對我有敵意  0 1 2 35. 自卑或覺得自己沒有價值  0 1 2 37. 常打架  0 1 2 38. 常被嘲笑  0 1 2 39. 常跟惹禍的同伴在一起  0 1 2 40. 聽到不存在的聲音  0 1 2 41. 衝動或不經考慮即行動  0 1 2 42. 喜歡獨處  0 1 2 43. 說謊或欺騙  0 1 2 45. 神經質或容易緊張  0 1 2 46. 出現緊張性動作或抽筋  0 1 2 48. 不被其他小孩喜歡  0 1 2 50. 過度害怕或焦慮  0 1 2 51. 覺得頭暈  0 1 2 52. 有過度的罪惡感  0 1 2 54. 過度疲倦  0 1 2 55. 體重過重  0 1 2 56. 有以下找不出生理原因的病痛：  0 1 2 a. 疼痛  0 1 2 b. 頭痛  0 1 2 c. 想吐，覺得身體不舒服  0 1 2 d. 與眼睛有關的問題  0 1 2 e. 發診或其他皮膚方面的問題  0 1 2 f. 胃吐或常說肚子痛  0 1 2 g. 嘔吐  0 1 2 57. 用身體攻擊他人  0 1 2 61. 功課不好  0 1 2 62. 動作協調不好或笨拙  0 1 2 63. 特別喜歡和年紀較大的孩子玩  0 1 2 64. 特別喜歡和年紀較小的孩子玩  0 1 2 65. 拒絕說話  0 1 2 66. 不斷重複某些動作；  0 1 2 67. 離家出走  0 1 2 68. 常大聲尖叫  0 1 2 69. 喜歡保守秘密，不希望別人知道我的事  0 1 2 70. 看到不存在的東西  0 1 2 71. 敏感，容易受窘  0 1 2 72. 縱火  0 1 2 74. 好賣弄或好扮小丑  0 1 2 75. 害羞或膽小  0 1 2 80. 會維護自己的權利  0 1 2 81. 在家裡偷東西  0 1 2 82. 在外面偷東西  0 1 2 84. 怪異的行為  0 1 2 85. 怪異的念頭  0 1 2 86. 倔強固執，悶悶不樂、煩躁易怒  0 1 2 87. 情緒突然的轉變  0 1 2 88. 喜歡和別人在一起  0 1 2 89. 多疑  0 1 2 90. 咒罵或說髒話  0 1 2 93. 太多話或愛說話  0 1 2 94. 常嘲笑別人  0 1 2 95. 脾氣暴躁  0 1 2 96. 太常想到性  0 1 2 97. 恐嚇他人  0 1 2 102. 活動量低、動作緩慢、或無精打采  0 1 2 103. 不快樂、悲傷，或沮喪  0 1 2 104. 說話異常大聲  0 1 2 105. 非醫療用途而使用酒精或麻醉品  0 1 2 106. 試著公平對待別人  0 1 2 112. 擔心、煩惱 | 0 1 2 1. I act too young for my age  0 1 2 3. I argue a lot  0 1 2 7. I brag  0 1 2 8. I have trouble concentrating or paying attention  0 1 2 9. I can’t get my mind off certain thoughts;  0 1 2 10. I have trouble sitting still  0 1 2 11. I’m too dependent on adults  0 1 2 12. I feel lonely  0 1 2 13. I feel confused or in a fog  0 1 2 14. I cry a lot  0 1 2 16. I am mean to others  0 1 2 17. I daydream a lot  0 1 2 19. I try to get a lot of attention  0 1 2 20. I destroy my own things  0 1 2 21. I destroy things belonging to others  0 1 2 22. I disobey my parents  0 1 2 23. I disobey at school  0 1 2 25. I don’t get along with other kids  0 1 2 26. I don’t feel guilty after doing something I shouldn’t  0 1 2 27. I am jealous of others  0 1 2 31. I am afraid I might think or do something bad  0 1 2 32. I feel that I have to be perfect  0 1 2 33. I feel that no one loves me  0 1 2 34. I feel that others are out to get me  0 1 2 35. I feel worthless or inferior  0 1 2 37. I get in many fights  0 1 2 38. I get teased a lot  0 1 2 39. I hang around with kids who get in trouble  0 1 2 40. I hear sounds or voices that other people think aren't there  0 1 2 41. I act without stopping to think  0 1 2 42. I would rather be alone than with others  0 1 2 43. I lie or cheat  0 1 2 45. I am nervous or tense  0 1 2 46. Parts of my body twitch or make nervous movements  0 1 2 48. I am not liked by other kids  0 1 2 50. I am too fearful or anxious  0 1 2 51. I feels dizzy  0 1 2 52. I feel too guilty  0 1 2 54. I fell overtired  0 1 2 55. I am overweight  0 1 2 56. Physical problems without known medical cause:  0 1 2 a. Aches or pains (not headaches)  0 1 2 b. Headaches  0 1 2 c. Nausea, feels sick  0 1 2 d. Problems with eyes  0 1 2 e. Rashes or other skin problems  0 1 2 f. Stomach aches  0 1 2 g. Vomiting, throwing up  0 1 2 57. I physically attack people  0 1 2 61. My school work is poor  0 1 2 62. I am poorly coordinated or clumsy  0 1 2 63. I would rather be with older kids than with kids my own age  0 1 2 64. I would rather be with younger kids than with kids my own age  0 1 2 65. I refuse to talk  0 1 2 66. I repeat certain acts over and over  0 1 2 67. I run away from home  0 1 2 68. I scream a lot  0 1 2 69. I am secretive or keep things to myself  0 1 2 70. I see things that other people think aren't there  0 1 2 71. I am self-conscious or easily embarrassed  0 1 2 72. I set fires  0 1 2 74. I show off or clown  0 1 2 75. I am shy  0 1 2 80. I stand up for my rights  0 1 2 81. I steal at home  0 1 2 82. I steal from places other than home  0 1 2 84. I do things other people think are strange  0 1 2 85. I have thoughts that other people would think are strange  0 1 2 86. I am stubborn  0 1 2 87. My moods or feelings change suddenly  0 1 2 88. I enjoy being with other people  0 1 2 89. I am suspicious  0 1 2 90. I swear or use dirty language  0 1 2 93. I talk too much  0 1 2 94. I tease others a lot  0 1 2 95. I have a hot temper  0 1 2 96. I think about sex too much  0 1 2 97. I threaten to hurt people  0 1 2 102. I don’t have much energy  0 1 2 103. I am unhappy, sad, or depressed  0 1 2 104. I am louder than other kids  0 1 2 105. I use alcohol or drugs for nonmedical purposes  0 1 2 106. I try to be fair to others  0 1 2 112. I worry a lot |
